# Sd-net: a semi-supervised double-cooperative network for liver segmentation from computed tomography (CT) images

**DOI:** 10.1007/s00432-023-05564-7

**Published:** 2024-02-05

**Authors:** Shixin Huang, Jiawei Luo, Yangning Ou, Wangjun shen, Yu Pang, Xixi Nie, Guo Zhang

**Affiliations:** 1 School of Communication and Information Engineering, Chongqing University of Posts and Telecommunications, Chongqing, 400065 China; 2 Department of Scientific Research, The People’s Hospital of Yubei District of Chongqing city, Chongqing, 401120 China; 3 West China Biomedical Big Data Center, West China Hospital, Chengdu, 610044 China; 4 School of Electrical and Electronic Engineering, Shanghai Institute of Technology, Shanghai, 201418 China; 5 Chongqing Human Resources Development Service Center, Chongqing, 400065 China; 6 School of Optoelectronic Engineering, Chongqing University of Posts and Telecommunications, Chongqing, 400065 China; 7 College of Computer Science and Technology, The Chongqing Key Laboratory of Image Cognition, Chongqing University of Posts and Telecommunications, Chongqing, 400065 China; 8 School of Medical Information and Engineering, Southwest Medical University, Luzhou, 646000 China

**Keywords:** Liver segmentation, Semi-supervised, Deep learning, Computed tomography (CT), Double-cooperative

## Abstract

**Introduction:**

The automatic segmentation of the liver is a crucial step in obtaining quantitative biomarkers for accurate clinical diagnosis and computer-aided decision support systems. This task is challenging due to the frequent presence of noise and sampling artifacts in computerized tomography (CT) images, as well as the complex background, variable shapes, and blurry boundaries of the liver. Standard segmentation of medical images based on full-supervised convolutional networks demands accurate dense annotations. Such a learning framework is built on laborious manual annotation with strict requirements for expertise, leading to insufficient high-quality labels.

**Methods:**

To overcome such limitation and exploit massive weakly labeled data, we relaxed the rigid labeling requirement and developed a semi-supervised double-cooperative network (SD- Net). SD-Net is trained to segment the complete liver volume from preoperative abdominal CT images by using limited labeled datasets and large-scale unlabeled datasets. Specifically, to enrich the diversity of unsupervised information, we construct SD-Net consisting of two collaborative network models. Within the supervised training module, we introduce an adaptive mask refinement approach. First, each of the two network models predicts the labeled dataset, after which adaptive mask refinement of the difference predictions is implemented to obtain more accurate liver segmentation results. In the unsupervised training module, a dynamic pseudo-label generation strategy is proposed. First each of the two models predicts unlabeled data and the better prediction is considered as pseudo-labeling before training.

**Results and discussion:**

Based on the experimental findings, the proposed method achieves a dice score exceeding 94%, indicating its high level of accuracy and its suitability for everyday clinical use.

## Introduction

In recent years, liver cancer has emerged as one of the most common and lethal forms of cancer worldwide, causing a large number of deaths each year (Ferlay et al. 2010) and seriously threatening people’s lives and health. Radiologists and oncologists study abnormalities in the form and texture of the liver by analyzing computed tomography (CT) or magnetic resonance images (MRI), which are commonly employed imaging modalities to analyze and diagnose the staging of liver lesions. These abnormalities are important biomarkers for early identification of primary and secondary liver malignancies and their progression (Lu et al. 2005; Moghbel et al. 2018). Diagnosis and treatment of liver cancer rely heavily on segmenting the liver from CT images to obtain liver volume data. CT image-based liver segmentation is the first and most critical step in any computerized technique for automatic detection of liver diseases, liver volume measurement and 3D liver volume rendering. Liver segmentation has many applications in clinical practice, such as radiomic analysis (Gillies et al. 2016), treatment planning (Rietzel et al. 2005), survival analysis (Zhang et al. 2020), and so on. Therefore, how to segment the liver region from abdominal CT images has become one of the hotspots in medical image segmentation (Fasihi and Mikhael 2016; He et al. 2008).

Segmenting the liver automatically from CT-enhanced images presents a formidable challenge (Ifty and Shajid 2023). This challenge arises due to several factors: 1 Low contrast and blurred edges: CT images often suffer from low contrast and blurred edges caused by partial volume effects resulting from spatial averaging, patient movement, beam hardening, and reconstruction artifacts. 2 Difficulty in extracting high gray levels: extracting regions with higher gray levels is particularly challenging because they are difficult to effectively separate from other gray levels. Additionally, distinguishing border regions in comparison to other gray levels is problematic. 3 Presence of similar-intensity organs: organs with similar intensity, such as the spleen, stomach, abdominal wall, and kidneys, are in close proximity to the liver. The exact spatial relationship of these neighboring organs with respect to the liver is often indistinct. These complexities underscore the need for the development of an analytical system that can perform fully automated liver segmentation in CT images.

Manual segmentation is an arduous and time-intensive task. This process can be expedited, streamlined, and made less susceptible to errors through the adoption of deep learning techniques. Image segmentation employing deep learning methods has garnered broad recognition for its resilience, efficiency, and reproducibility. Recently, deep neural networks have obtained impressive progress for automatic liver tumor segmentation (Christ et al. 2016; Ben-Cohen et al. 2016; Li et al. 2018; Zhang et al. 2019). However, these leading approaches rely on accurate pixel-wise annotations. Obtaining such annotations is very difficult because it is time-consuming and has strict demands for expertise. Therefore, it is desirable to develop deep learning methods which can work well when high-quality labeled data is not available.

To cope with these challenges, we proposed a semi-supervised double-cooperative network (SD-Net) that is able to utilize a large number of unlabeled or weakly labeled datasets to compensate for sparse densely labeled datasets. This framework comprises two collaborative network models, VNet and 3D-ResVnet. In the supervised training module, an adaptive mask fine-tuning is proposed. Two network models are first used to predict the labeled dataset separately, and then adaptive mask refinement is applied to the difference predictions to obtain more accurate liver segmentation results. In the unsupervised training module, a dynamic pseudo-label generation is proposed. First, the two models each predict the unlabeled data, and the model with better prediction results is considered as pseudo-labeled for subsequent training. Liver segmentation experiments on the LiTS dataset verify that the proposed SD-Net has state-of-the-art performance, approximating the performance of the fully supervised method.

The main contributions of this paper are summarized as follows.

- We propose a novel semi-supervised Double-cooperative framework for liver segmentation that involves two collaborative network models. This approach relaxes the rigid labeling requirements commonly associated with supervised convolutional networks, allowing for the exploitation of massive weakly labeled data.
- To improve the segmentation accuracy, we propose an adaptive mask fine-tuning that rechecks the region of difference between the two model predictions, resulting in a more accurate liver segmentation.
- We propose a dynamic pseudo-label generation strategy that leverages the better predicted masks from both network models as pseudo-labels, thereby enhancing the quality of these labels for the unsupervised training module.
- The experimental results of our research demonstrate a dice score exceeding 94%, affirming the high level of accuracy and the clinical suitability of our method.

The remainder of this paper is organized as follows: Section "Related work" briefly reviews manual and deep learning, as well as pseudo-labeling-based and semi-supervised liver segmentation methods. Section "Methodlogy" describes the principle and framework implementation of SD-Net. Experiments and analysis are given in Section "Experiments and Results". Section "Conclusion" concludes the paper.

## Related work

### Hand-crafted feature based methods

In order to solve the problem of liver CT image segmentation, many methods have been proposed by experts and researchers. Traditional liver segmentation methods are categorized into: intensity threshold (Lim et al. 2006; Soler et al. 2001), region growing (Ruskó et al. 2007; Pohle and Tönnies 2001), and deformable model (Kainmüller et al. 2007; Park et al. 2003).

Intensity thresholding based segmentation is used to segment liver and non-liver regions with fixed or adaptive thresholds of gray values or other features of the image. (Liu and Chen 2015) proposed an algorithm for intrahepatic vessel segmentation based on two-stage region growth. However, this method not only requires manual selection of seed points to determine the liver area, but also relies on the threshold value in the preset growth rule. This method is simple but may not be accurate enough for complex situations.

The region growing based method grows the segmented region by merging the similarities of neighboring pixels starting from the seed point. (Lee et al. 2007) proposed a fast liver segmentation method for CT images. First seed region growing is applied to horizontal set velocity images to detect the initial boundary of the liver, and then a rolling ball algorithm is used to refine the liver boundary more accurately. (Suzuki et al. 2010) proposed a liver extraction method based on a combination of geodesic activity contour segmentation and level set contour evolution. The method first performs anisotropic diffusion filtering on CT images and enhances the liver boundary using scale-specific gradient magnitude filtering. Then a fast-marching level-set algorithm is used to generate an initial contour of the liver. Finally the initial contour is refined by combining the geodesic active contour segmentation algorithm for level-set contour evolution to de-calculate the liver volume. This method is sensitive to noise, but works well in some situations.

Deformable model based segmentation is a commonly used method for medical image segmentation that allows automatic adaptation of the model shape based on image features. (Chen et al. 2012) proposed a liver 3D segmentation method based on an improved active appearance model and combining live wire and graph cuts strategies. The method first constructs the model, then adopts a pseudo-3D initialization strategy on the realization of segmenting the organ slice by slice, and finally proposes the always-3D shape constraint method to segment the target. (Kainmüller et al. 2007) proposed a fully automated 3D segmentation method of liver based on CT data, which is mainly based on the combination of constrained free-form variational model and statistical deformation model, and designed the displacement force calculation and parameter estimation to solve the liver segmentation problem. However, these methods require some manual marking of points, rely on hand-crafted features, and have limited feature representation capabilities.

### Deep learning based methods

Compared to traditional methods, deep learning-based liver segmentation method is a data-driven approach (Furqan Qadri et al. 2019; Qadri et al. 2019, 2021) that allows end-to-end optimization without manual feature engineering (Litjens et al. 2017). Many of the early deep learning-based liver segmentation methods combined neural networks with specialized post-processing routines. (Christ et al. 2016) used 3D fully connected neural networks combined with dense 3D conditional random field. (Hu et al. 2016) proposed a framework for automatic liver segmentation based on 3D convolutional neural networks (CNNs) and globally optimized surface evolution. First, a 3D CNN is trained to give an initial surface as a shape prior for the segmentation step, and then the prior segmentation is fused into the segmentation model. (Lu et al. 2017) proposed a deep learning algorithm with graph cut refinement to automatically segment the liver. First, liver detection and probabilistic segmentation are performed simultaneously using a 3D convolutional neural network. Then, the initial segmentation is precisely refined using graph cuts and previously learned probabilistic graphs. U-Net derived architectures are heavily exploited in liver segmentation. (Ifty and Shajid 2023) proposed a liver segmentation model based on U-Net. (Ansari et al. 2022) proposed a method utilizing fixed-width residual UNet skeleton and pyramidal cavity convolution. To further improve the performance, (Kavur et al. 2022) proposed to combine four neural networks, U-Net, Deepmedic, V-Net, and Dense V-Networks. (Xie et al. 2022) proposed a multi-scale context integration network, which utilizes residual modules to prevent network degradation, as well as cascading to capture broad and deeper features. (Ahmad et al. 2019a) proposed a deep belief network by unsupervised pretraining and supervised fine-tuning. Furthermore, (Ahmad and Syed 2022) proposed a lightweight convolutional neural network for liver segmentation, which greatly reduced the training time.

Deep learning based liver segmentation methods (Ahmad et al. 2018, 2019b) can improve the automation of the image segmentation process, which can greatly save time and effort, eliminate human subjectivity, and improve segmentation accuracy.

### Pseudo-labeling methods

Pseudo-labeling is a technique employed in deep neural networks during semi-supervised training. In the context of semi-supervised training, the objective is to produce pseudo-labels for unlabeled data, and the key consideration revolves around the generation of trustworthy pseudo-labels. (Lee 2013) represents one of the initial investigations into semi-supervised learning utilizing pseudo-labels. In this research, unlabeled samples with high confidence for pseudo-labeling are directly chosen using a static threshold. A more streamlined approach, known as FixMath (Sohn et al. 2020), begins by predicting pseudo-labels for moderately improved unlabeled images, retaining only those with high-confidence pseudo-labels. Subsequently, it predicts pseudo-labels for strongly enhanced iterations of the same images. Nonetheless, this approach relies on a fixed, pre-defined threshold applicable to all categories when selecting unlabeled data for training, without accounting for varying learning conditions and challenges across different categories. In addressing this limitation, (Zhang et al. 2021) introduced Flexmatch, a method that leverages unlabeled data based on the model’s learning dynamics, dynamically adjusting category-specific thresholds at each time step. The disparity in distribution between the labeled and unlabeled datasets introduces substantial biases in semi-supervised learning pseudo-labels, leading to a notable decline in performance. In order to mitigate this issue, (Zhao et al. 2022) introduce Distributive Consistent Semi-Supervised Learning, which involves the direct estimation of a reference class distribution and subsequently enhances the pseudo-labels by promoting a gradual convergence of the predicted class distribution of unlabeled data towards the reference class distribution.

### Semi-supervised medical image segmentation methods

The acquisition of top-notch labeled medical image data poses difficulties due to the necessity of annotations by experienced radiologists. This hurdle serves as a catalyst for the advancement and investigation of semi-supervised methods in medical image segmentation. Wu et al. (2021); Yao et al. (2022) is dedicated to the generation of dependable pseudo-labels, whereas (Li et al. 2020; Chen et al. 2022; Luo et al. 2021; Xie et al. 2021) delves into the utilization of consistency regularization.

## Methodology

Segmenting the liver in a medical image involves the task of pinpointing a cluster of voxels that best represent the anatomical region occupied by the liver. However medical images are challenging to acquire high quality labeled data as they need to be annotated by experienced radiologists. To this end, we propose the SD-Net for liver segmentation that learn to transfer from the source domain of a labeled CT image to the unlabeled target domain.

**Fig. 1:**
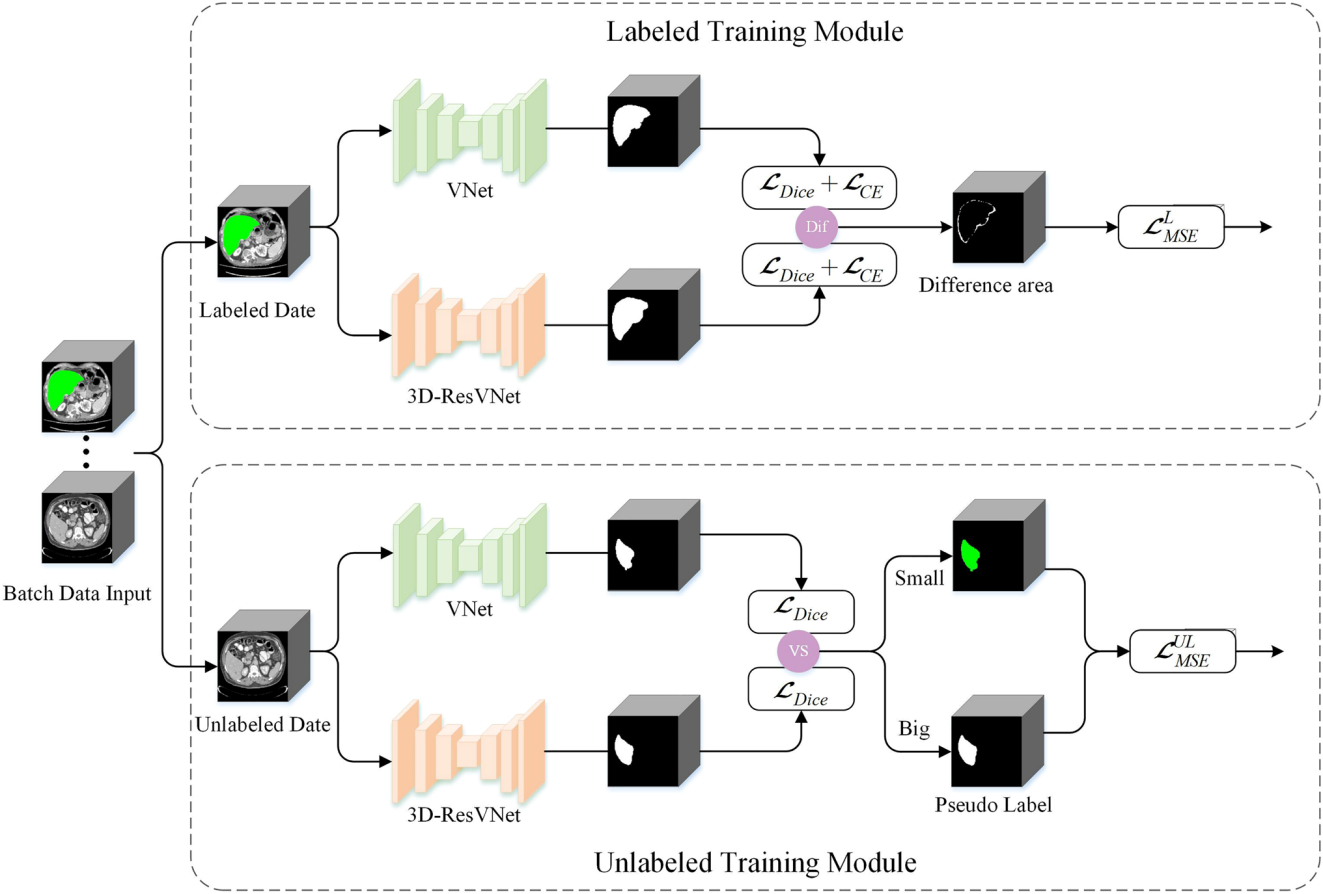
Illustration of the SD-Net including adaptive mask fine-tuning and dynamic pseudo-label generation

The training procedure of the proposed method is shown in Fig. 1. We chose two sub-networks with comparable performance, VNet and 3D-ResVNet, being defined as $f_{{{\text{VNet}}}} ( \cdot )$ and $f_{{{\text{3D - ResVNet}}}} ( \cdot )$, respectively. In semi-supervised scenario, a set of m label data is given with corresponding datasets $D_{{{\text{Label}}}} = \{ D^{1} ,D^{2} ,D^{3} ,...,D^{m} \}$ where contains $N_L$ image/label pairs denoted as $D^L=\{(x_i^L, g_i^L)\}_{i=1}^M$, and n unlabeled datasets $D_{{{\text{Unlabel}}}} = \{ D^{{m + 1}} ,D^{{m + 2}} ,D^{{m + 3}} ,...,D^{{m + n}} \}$ contains $N_U$ images denoted as $D^U=\{(x_i^U)\}_{i=1}^{M+N}$ (usually $N\le M$). ${{x}_{i}}\in {{R}^{H\times W\times D}}$ is liver volume and ${{g}_{i}}\in {{\{0,1\}}^{H\times W\times D}}$ is the ground-truth label. A batch of input data *X* includes equal proportions of labeled $(X^L,G^L)$ and unlabeled data $X^U$, and liver volumes are sent to VNet and 3D-ResVNet:

1 $$\hat{G}_{{{\text{VNet}}}}^{{\text{L}}} ,\hat{G}_{{{\text{VNet}}}}^{{\text{U}}} = f_{{{\text{VNet}}}} (X)$$

2 $$\hat{G}_{{{\text{3D - ResVNet}}}}^{{\text{L}}} ,\hat{G}_{{{\text{3D - ResVNet}}}}^{{\text{U}}} = f_{{{\text{3D }} - {\text{ResVNet}}}} (X)$$

The outputs include labeled and unlabeled liver volumes predictions: $\hat{G}=\hat{G}^L \cup \hat{G}^U$. For labeled data to predict $\hat{G}^L$, we use the supervised loss function ${\mathcal {L}}_{s}$. For unlabeled data to predict $\hat{G}^U$, we adopt the unsupervised loss function ${\mathcal {L}}_{uns}$, which generates dynamic pseudo-labels. The proposed SD-Net employ both networks, taking full advantage of their strengths.

### Adaptive mask fine-tuning

In label training module, we designed a refined network framework. In the segmentation task, when using different backbones, the predicted segmentation results are usually inconsistent, and one of the models must be wrongly predicted. For this reason, we designed two backbones to predict the liver segmentation, considering the region where both predictions are the same as the correct segmentation region, and the region where they are not the same as the uncertain segmentation region. For the uncertain region, we use an MSE loss function to constrain it again. The uncertain region is defined as:

3 $$G_{{{\text{dif}}}}^{{\text{L}}} = {\text{Difference}}\left( {\hat{G}_{{{\text{VNet}}}}^{{\text{L}}} ,\hat{G}_{{{\text{3D - ResVNet}}}}^{{\text{L}}} } \right)$$

where $Difference(\cdot )$ denotes an operation to obtain the difference between the masked regions predicted by the two backbone.

**Algorithm 1:**
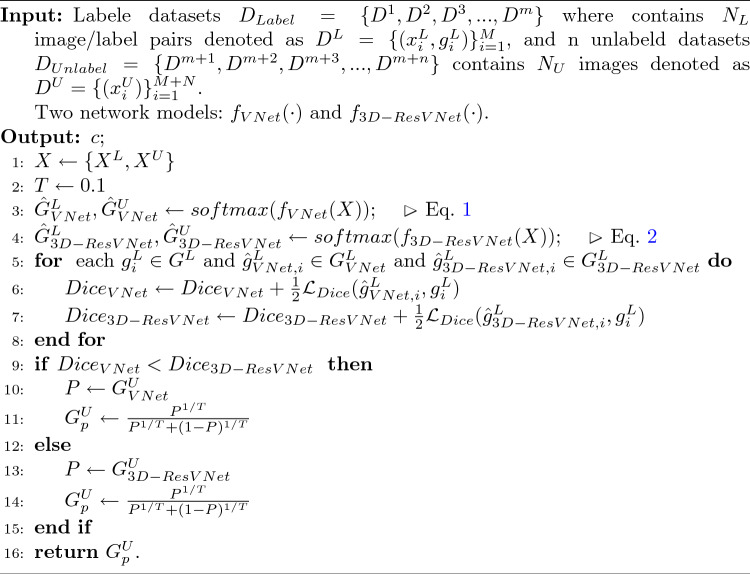
Dynamic pseudo-label generation

### Dynamic pseudo-label generation

In unlabel training module, we propose a dynamic pseudo-label generation method, and the detailed algorithm flow is shown in Algorithm 1. We directly employ the Dice loss to assess the real-time segmentation performance of two models. By comparing the loss values computed on the labeled dataset, we select the model with the smaller loss value to serve as the pseudo-label generator for the model with the larger loss value. Following entropy minimization, the network predictions are transformed into soft pseudo-labels using the sharpening function (Xie et al. 2020). Since we use the Dice loss as a criterion for predicting segmentation performance, which can directly reflect the dice coefficients, no additional computation is introduced.

### Loss function

Loss function is used to measure the performance of the model and help the model to improve during the training process. Cross entropy loss and Dice loss (Drozdzal et al. 2016) are the two most commonly used loss functions for image segmentation tasks. The cross-entropy loss is defined as:

4 $$\begin{aligned} {{\mathcal {L}}_{CE}}({{\hat{G}}^{L}},{{G}^{L}})=-\frac{1}{M}\sum \limits _{l=1}^{L}{\sum \limits _{m=1}^{M}{G_{m}^{L}\log \hat{G}_{m}^{L}}} \end{aligned}$$

where $G_{m}^{L}$ is the ground truth binary indicator of class label *L* of *m*. $\hat{G}_{m}^{L}$ is the corresponding predicted segmentation probability.

Dice loss is to subtract the dice score from 1 to get an amount that needs to be minimized. Thus, class imbalance can be implicitly incorporated into the learning process without explicitly introducing class-specific weights or other class rebalancing techniques.

5 $$\begin{aligned} {{\mathcal {L}}_{Dice}}({{\hat{G}}^{L}},{{G}^{L}})=1-\frac{2\sum \limits _{l=1}^{L}{\sum \limits _{m=1}^{M}{G_{m}^{L}\log \hat{G}_{m}^{L}}}}{\sum \limits _{l=1}^{L}{\sum \limits _{m=1}^{M}{G_{m}^{L}}} +\sum \limits _{l=1}^{L}{\sum \limits _{m=1}^{M}{\hat{G}_{m}^{L}}}} \end{aligned}$$

*In Label Train Module,* the default loss function is the unweighted sum ${\mathcal {L}}_{CE}+{\mathcal {L}}_{Dice}$. We employ MSE loss to guide the model to review these potentially mispredicted areas, which is as follows:

6 $$\begin{aligned} {{\mathcal {L}}_{MSE}^{L}}({{\hat{G}}_{dif}^{L}},{{G}_{dif}^{L}})= -\frac{1}{n}\sum \limits _{n=1}^{N}{{{(G_{dif}^{L}-\hat{G}_{dif}^{L})}^{2}}} \end{aligned}$$

To this end, supervisory loss contains ${\mathcal {L}}_{CE}$, ${\mathcal {L}}_{Dice}$ and ${\mathcal {L}}_{MSE}^{L}$, which is defined as:

7 $$\begin{aligned} {\mathcal {L}}_{s}={\mathcal {L}}_{CE}+{\mathcal {L}}_{Dice}+{\mathcal {L}}_{MSE}^{L} \end{aligned}$$

*In Unlabel Train Module,* the unsupervised MSE loss is adopted, which is defined as:

8 $$\begin{aligned} {\mathcal {L}}_{uns}={{\mathcal {L}}_{MSE}^{UL}}({{\hat{G}}^{U}},{{G}^{PL}})= -\frac{1}{n}\sum \limits _{n=1}^{N}{{{(G_{n}^{PL}-\hat{G}_{n}^{U})}^{2}}} \end{aligned}$$

where ${G}^{PL}$ is pseudo label.

## Experiments and results

### Experimental setup

#### Datasets

The LiTS dataset (Bilic 2023) contains 201 contrast-enhanced 3D abdominal CT images, where 194 CT scans contain lesions. The dataset was acquired from seven different scanners and scanning protocols from clinical sites around the world, with in-plane image resolution ranging from 0.56 mm to 1.0 mm and slice thickness ranging from 0.45 mm to 6.0 mm. Additionally, the minimum number of axial slices in the CT scans was 74, while the maximum number of slices was 987. We split the dataset into 104 volumes for training, 26 volumes for validation and 70 volumes for testing, using liver volumes that were not significantly different. Tumor masks are provided for the training dataset, while the ground truth data for the testing dataset is withheld for online validation. For image preprocessing, the CT image intensity values were truncated to a range of [0, 400] Hounsfield units (HU) to remove irrelevant details.

#### Evaluation metrics

To assess the performance of the model, we use a Dice per case score and Dice global score to assess the whole liver and tumor segmentation performance, as well as specificity, sensitivity, accuracy, Jaccard. The Dice per case score represents an average Dice score calculated for each individual volume or case, and the Dice global score refers to the Dice score calculated on a unified dataset where all scans are amalgamated or merged together.

Dice score (Dice 1945) is used as a performance metric for evaluating the model predictions that serves to gauge the resemblance between two images. It computes the F1 score, a value derived from the harmonic mean of recall and precision. In this particular context, it finds application in binary pixel classification. When confronted with binary segmentation tasks, Dice score assesses the extent of overlap between the ground truth mask *G* and the predicted segmentation mask *P* which is calculated as follows:

9 $${\text{Dice}}(G,P) = \frac{{2\left| {G \cap P} \right|}}{{\left| G \right| + \left| P \right|}}$$

Dice scores in the interval [0, 1] with no defective segmentation results scored as 1.

In liver segmentation, high sensitivity means that the model is more able to capture liver regions correctly and avoid missing truly positive regions. This is important to ensure that liver tissue is detected as accurately as possible.

10 $${\text{Sensitivity}} = \frac{{{\text{TP}}}}{{{\text{TP + FN}}}}$$

where *TP* is the number of true positives and *FN* is the number of false negatives.

The level of specificity relates to the model’s ability to label other structures as liver without error. High specificity reduces the risk of incorrectly labeling non-liver regions as liver.

11 $${\text{Specificity}} = \frac{{{\text{TN}}}}{{{\text{TP + FP}}}}$$

where *TN* is the number of true negatives and *FP* is the number of false positives.

Accuracy can provide an overall assessment, considering both true positives and true negatives.

12 $${\text{Accuracy}} = \frac{{{\text{TP + TN}}}}{{{\text{TP + TN + FP + FN}}}}$$

In liver segmentation, the Jaccard index (Jaccard 1912) can provide information about the overlap between model predictions and actual labeling. High Jaccard indices indicate that the model predictions are more similar to the actual segmentation.

13 $${\text{Jaccard}} = \frac{{{\text{TP}}}}{{{\text{TP + FP + FN}}}}$$

#### Implementation details

**Fig. 2:**
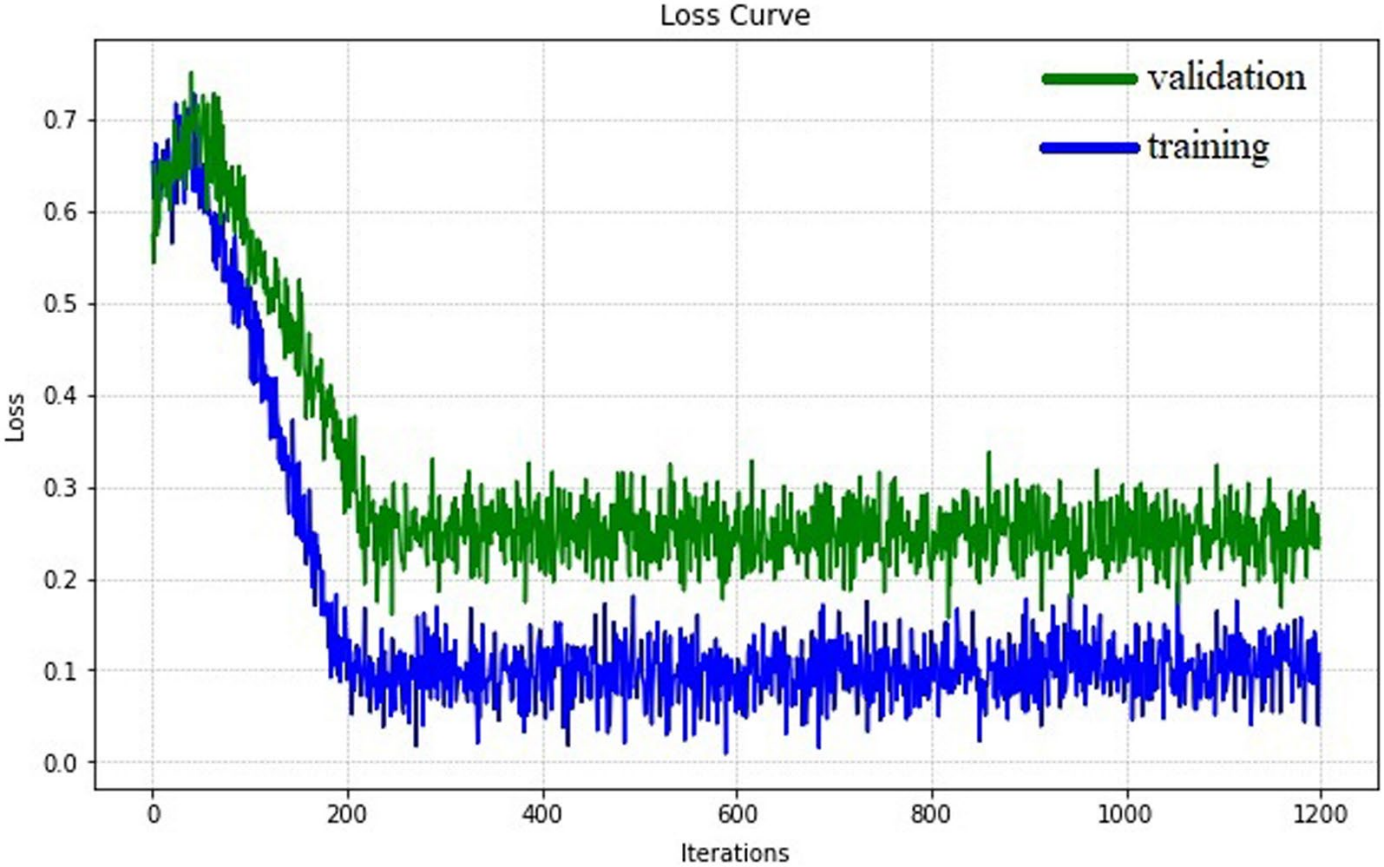
Training and validation dice loss against iterations

We deploy the SD-Net model on NVIDIA V100 GPUs and use PyTorch as the implementation platform. In order to better display the liver region, the original CT image was window width is set to 400 and window position is set to 0. To expand the dataset, the input data is randomly flipped and rotated during the training process machine flipping and rotating. In particular, we optimize using stochastic gradient descent (SGD), where the weight decay is 0.0001 and momentum is 0.9. The initial learning rate is set to 0.01 and divided by 10 after every 200 iterations, for a total of 1200 iterations. The training batch was 4, of which 2 were labeled data volumes and the other 2 were unlabeled volumes. The variation of training dice loss (blue line) and validation dice loss (green line) is shown in Fig. 2. It can be observed that the loss stabilizes after the model is trained to 200 iterations.

### Comparison experiments

Our model is compared with other state-of-the-art methods, including MS-Net (Shah et al. 2018), MSDN (Wang et al. 2019), SCN (Ibrahim et al. 2020), DS-ResUnet (Zhang and Zhang 2020) and (Sun et al. 2020), to verify the superiority in segmentation accuracy. Out of these 5 comparison methods, the full supervision method is the only one, called DS-ResUnet. Deep learning-based medical image segmentation models often necessitate large datasets containing high-quality dense segmentations for training. Preparing such datasets can be extremely time-consuming and expensive. Addressing this challenge, the mixed-supervised dual-network (MSDN) (Wang et al. 2019) is proposed where only a portion of the data is densely labeled with segmentation labels while the rest is weakly labeled with bounding boxes. MS-Net (Shah et al. 2018) is a new FCN that combines strong and weak supervision, thus significantly reducing the supervision cost. SCN (Ibrahim et al. 2020) is a semi-supervised framework that uses only a small set of fully supervised images and a set of images labeled only with object bounding boxes. The framework trains a master segmentation model with the help of an auxiliary model that generates initial segmentation labels for the weak set and a self-correcting module that improves the generated labels during training using the master model with increasing accuracy. SAM (Kirillov et al. 2023) combines these two sources of information from the image encoder and prompt encoder into a lightweight mask decoder that de-segments the mask. Zhang and Zhang (2020) proposed a deeply supervised residual Unet (DS-ResUnet) for fully automated segmentation of the liver region in abdominal enhanced CT images. The following is a quantitative and qualitative analysis of the comparison methods.

#### Quantitative evaluation

Table 1 demonstrates the segmentation performance using dice global, dice per case, sensitivity, specificity, accuracy, and Jaccard as evaluation metrics. It can be seen that the proposed method outperforms the unsupervised methods of MSDN, MS-Net, SCN, SAM and Sun et al. (2020), and achieves the highest segmentation accuracy among similar models, approximating the fully supervised method DS-ResUnet. Although the performance of the proposed SD-Net is relatively poorer compared to that of the fully supervised DS-ResUnet, it utilizes fewer labeled data.

**Table 1:** Comparison of the non-fully supervised models MSDN, MS-Net, SCN, SAM and Sun et al. (2020) with our proposed SD-Net and the fully supervised model DS-ResUnet in terms of objective metrics

Methods	Metrics (%)					
	Dice global	Dice per case	Specificity	Sensitivity	Accuracy	Jaccard
MSDN Wang et al. (2019)	86.7	87.1	94.44	84.07	90.70	76.53
MS-Net Shah et al. (2018)	90.8	90.5	96.65	88.63	93.95	83.16
SCN Ibrahim et al. (2020)	92.6	92.6	97.79	89.47	94.72	86.22
Sun et al. (2020)	92.8	93.1	97.62	90.14	94.88	86.58
SAM Kirillov et al. (2023)	93.54	93.32	97.00	88.91	94.26	83.98
DS-ResUnet Zhang and Zhang (2020)	96.06	95.08	96.06	–	96.11	92.54
Ours	94.53	94.12	97.03	95.08	96.40	89.63

The dice coefficient is expressed in percentiles

#### Qualitative evaluation

**Fig. 3:**
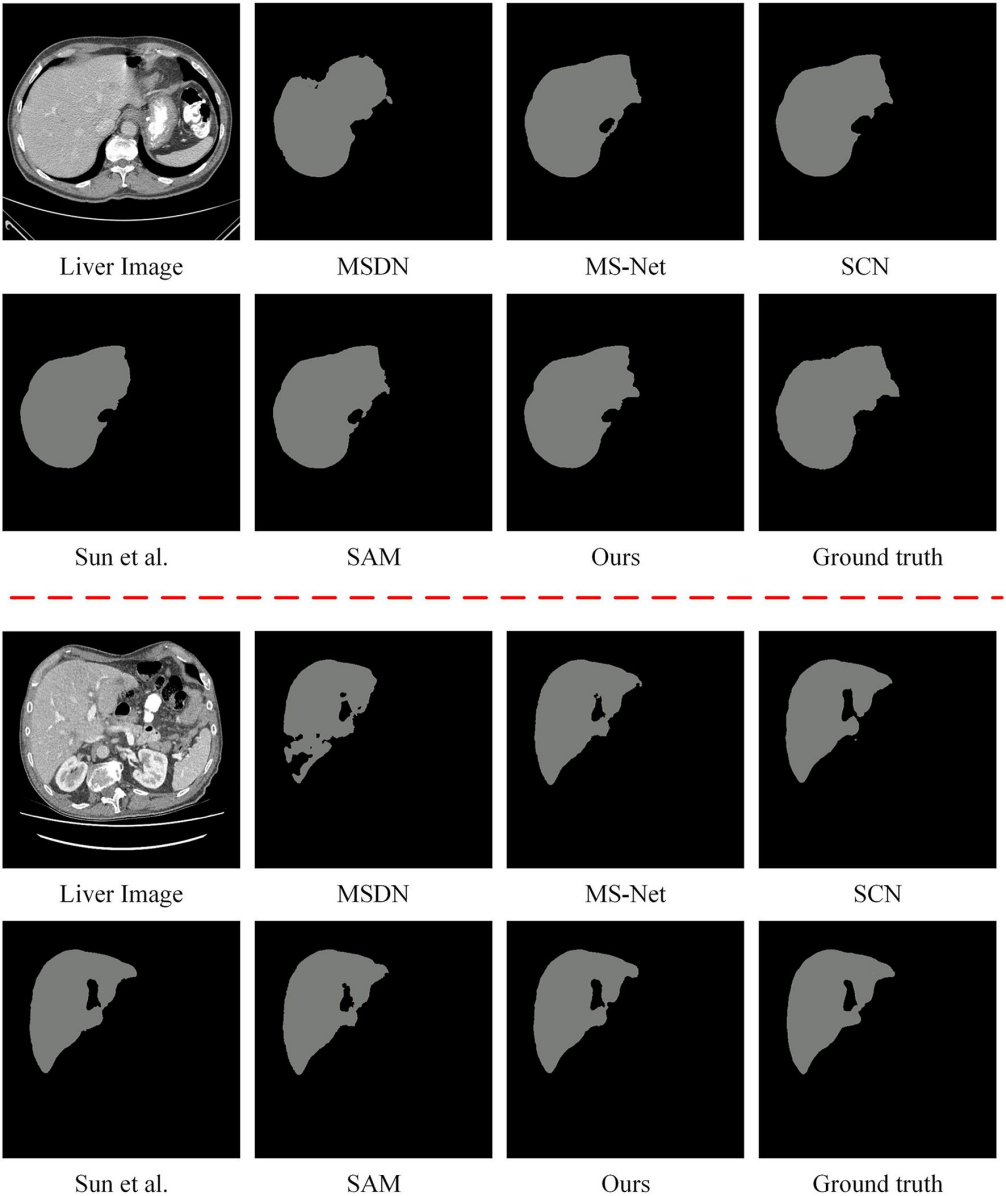
The segmentation results of the comparison methods

Enhanced CT liver region segmentation results of the comparison methods are shown in Fig. 3. Notably, the segmentation results of DS-ResUnet are not shown because its code is not provided. From Fig. 3, it can be seen that the MSDN’s segmented out structural boundaries are fractured with obvious jagged boundaries. In addition, there are more Disconnected Regions (DRs), which cannot well maintain the integrity of the liver morphology. The segmentation results of SCN were not smooth enough at the edges,and the problem of under-segmentation occurred, resulting in insufficient details of the edge structure. In contrast, the liver segmentation results of MS-Net and Sun et al. (2020) have smoother and more coherent boundaries. However, the segmentation boundaries of MS-Net are substantially offset from the true value boundaries and cannot accurately outline the target structure, which may be caused by its segmentation algorithm’s over-tolerance of weak boundaries. The segmentation result of Sun et al. (2020) has a noise region that is obviously segmented out by mistake, which causes the problem of over-segmentation and fails to maintain the liver morphology effectively. In contrast to the shape and color of regular objects in natural images, the overall texture of tissues in medical images is much sparser and more homogeneous, resulting in the inability of SAM (Kirillov et al. 2023) to accurately outline the liver structure. The segmentation results of the proposed method have a higher degree of overlap with the ground truth, basically preserving the morphology of the structure and the smooth coherence of the boundary. Therefore, the qualitative results verify that the proposed method has the most accurate segmentation results, in which it outperforms the other methods in terms of boundary smoothness, boundary offset, the degree of overlap of segmented real organs, and contour integrity.

### Ablation

#### Ablation for loss

The difference between areas obtained from the predictions of both VNet and 3D-ResVNet networks are very small and scattered. For this reason, we try to use the common MSE Loss and Weighted Cross-Entropy Loss for ablation experiments. Table 2 shows the segmentation performance of the model using both losses where it can be seen that the segmentation is better using MSE Loss. To this end, we use MSE Loss ${\mathcal {L}}_{MSE}^{L}$ in the labeled training module.

**Table 2:** Performance comparison using different losses

Loss function	Metrics	
	Dice global (%)	Dice per case (%)
Weighted cross-entropy loss	94.32	93.76
MSE Loss	94.53	94.12

#### Ablation for number of iterations

**Fig. 4:**
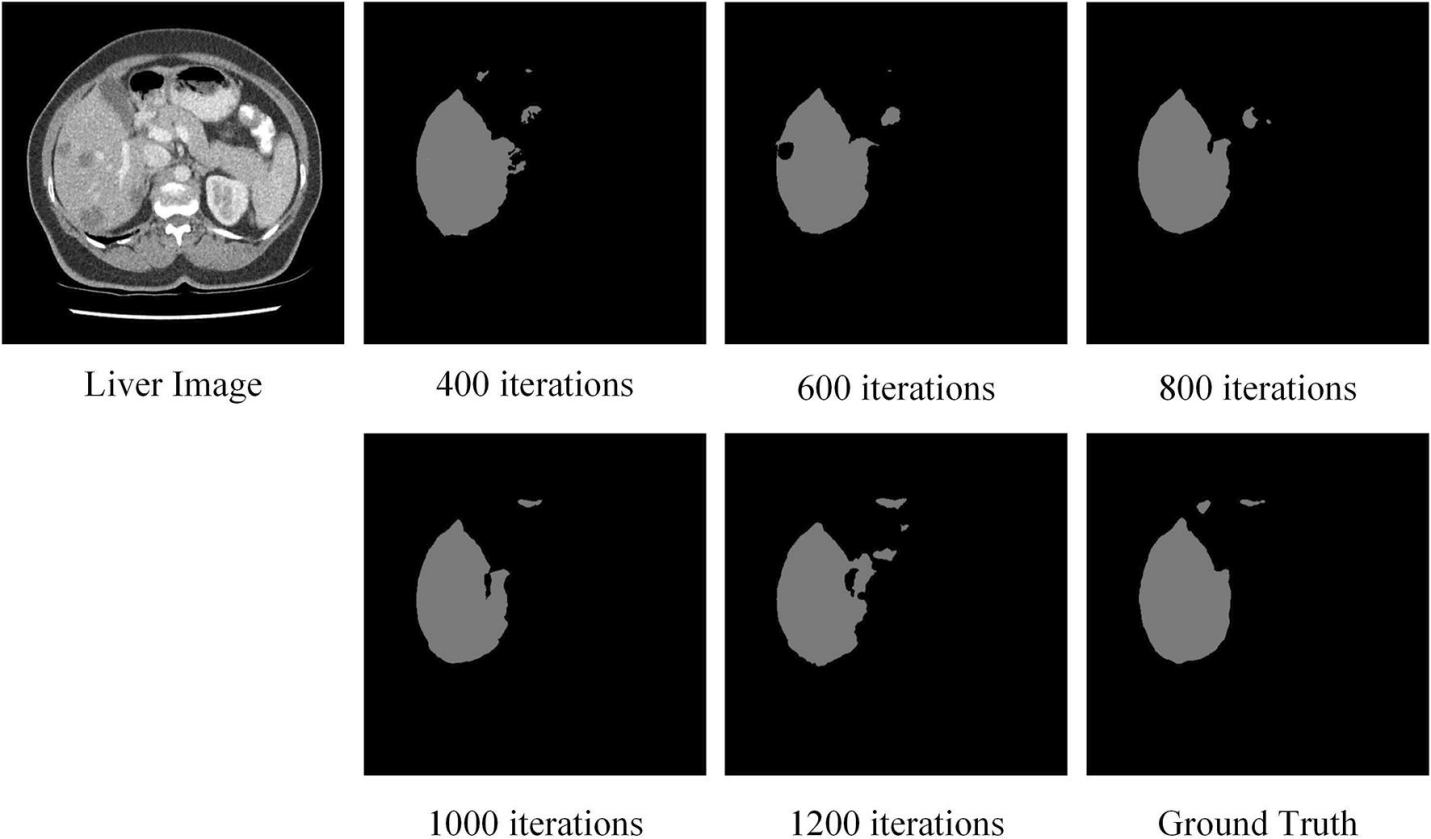
The segmentation results obtained for different number of training iterations

When segmenting the liver training, the model usually stabilizes after 200 iterations. The segmentation results obtained for different number of training iterations are shown in Fig. 4. It can be seen that the liver segmentation at 600 and 1200 iterations has more Disconnected Regions, the segmentation effect is too fragmented, and the wholeness of the liver morphology is poorly maintained. The segmentation results at 400 and 800 iterations cannot accurately outline the target structure. At 1000 iterations, the proposed SD-Net obtains the best segmentation results. Therefore, we chose the training model with 1000 iterations.

### Analysis of the ratio of strong and weak datasets

**Fig. 5:**
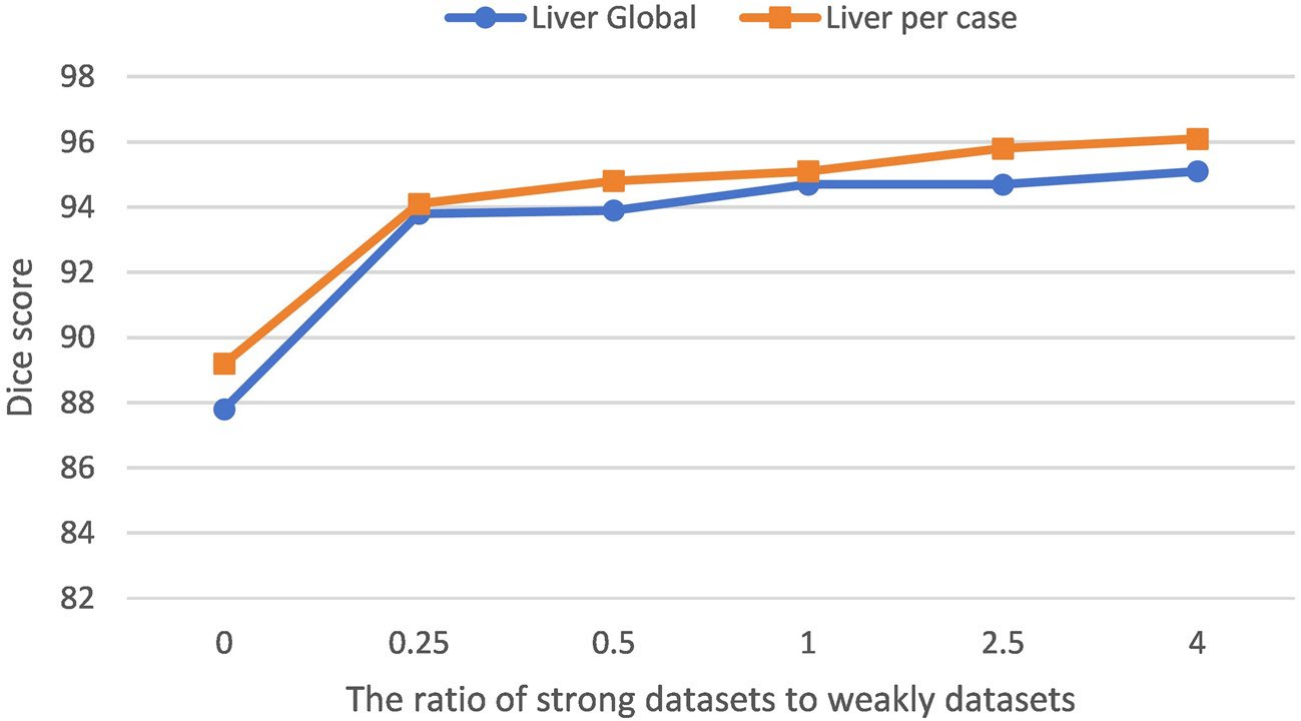
Performance variation of the proposed SD-Net with the ratio of strong datasets to weak datasets

**Fig. 6:**
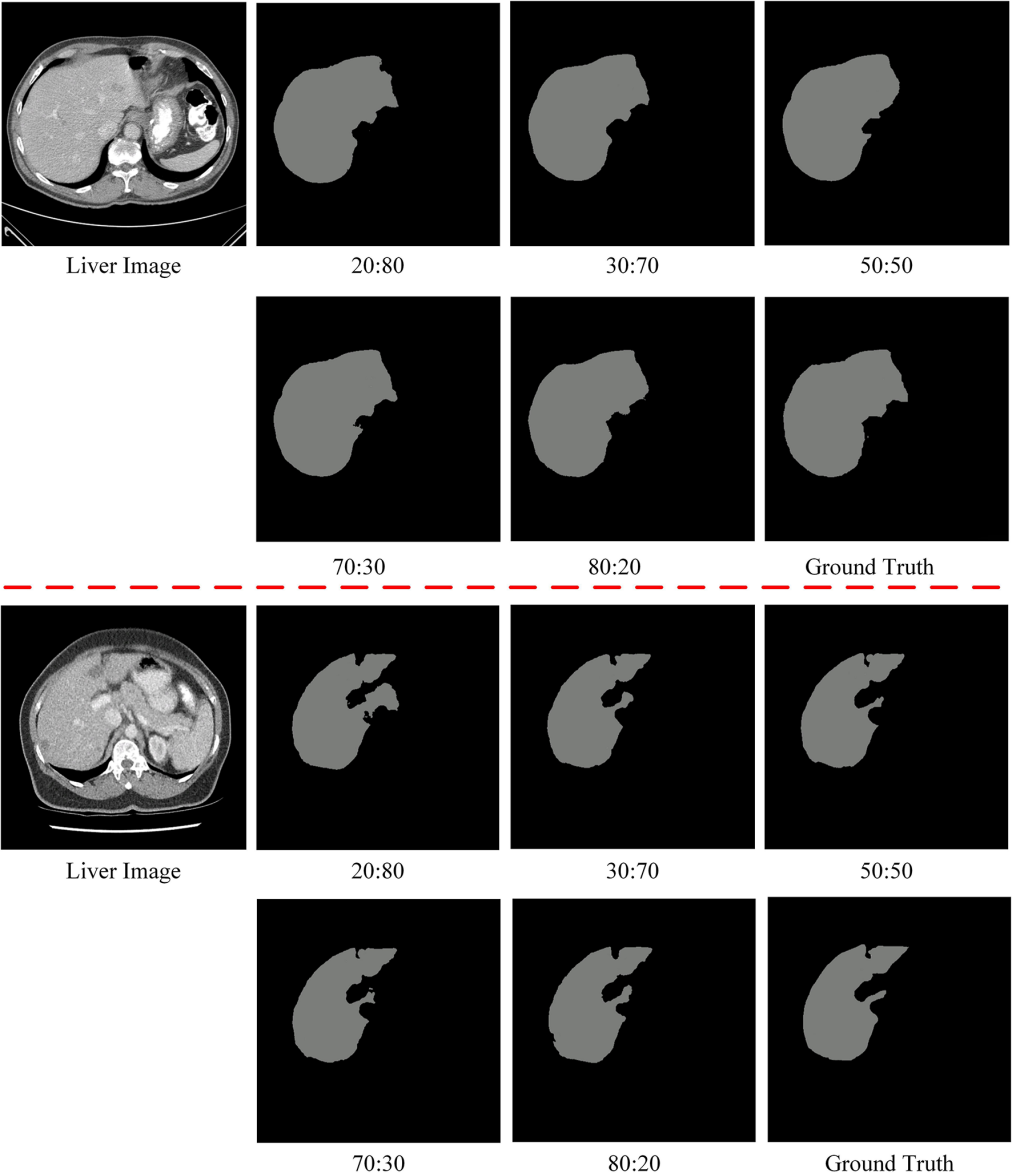
The segmentation results under different raito of strong and weak datasets

We will validate the proposed model based on different percentages of strong and weak datasets. Theoretically, a higher percentage of strong dataset indicates that there are more labeled data in the training and it is closer to intensive supervised training. Among the 131 split-labeled public scans in the LiTS training dataset, in which 31 scans are reserved for testing and 100 scans are used for training. We design the training dataset to be split into strong and weak datasets in the ratio of 20:80, 30:70, 50:50, 70:30, and 80:20. We use Dice global and Dice per case as evaluation metrics. Note that we also design a fully supervised ratio of 100:0, which means that all training data is labeled. In Fig. 5, it is demonstrated that the performance of the model varies with the proportion of strong and weak datasets. It can be seen that the proposed SD-Net achieves a segmentation performance of more than 94% for the 20:80 ratio and increases as the proportion of strong datasets increases. Figure 6 shows the segmentation results of different ratios of strong and weak datasets, and it can be seen that the proposed SD-Net still retains the contour of the liver region under the 20:80 ratio, which is close to the ground truth.

## Conclusion

In this paper, we present a SD-Net learning framework for liver segmentation that relaxes the requirement of dense labeling. The framework introduces VNet and 3D-ResVNet network models, and updates the parameters independently to play the potential of the two networks. Adaptive mask fine-tuning is to re-examine the difference regions predicted by the two network models, which can improve the segmentation accuracy of the liver. Dynamic pseudo-label generation is to use the better predicted masks from both network models as pseudo-labels to improve the quality of pseudo-labels. The experimental results of liver segmentation on segmented dataset show that the proposed semi-supervised double-cooperative framework has state-of-the-art performance, and our model achieves comparable performance compared to the fully supervised strategy. It also demonstrates the potential of the proposed method to be applied in real clinical practice.

## Data Availability

LiTS17 is a liver tumor segmentation benchmark (https://competitions.codalab.org/competitions/17094). The data and segmentations are provided by various clinical sites around the world.
